# External Morphology of *Leporinus oliveirai* (Characiformes: Anostomidae) During Early Life Stages

**DOI:** 10.1002/jmor.70065

**Published:** 2025-07-11

**Authors:** Ian Solon Bortoloci Ito, Mariana Pascoal Boaretto, Marcos Venturieri, Andréa Bialetzki, José Luís Olivan Birindelli

**Affiliations:** ^1^ Programa de Pós‐Graduação em Ciências Biológicas, Departamento de Biologia Animal e Vegetal Universidade Estadual de Londrina Londrina Brazil; ^2^ Piscicultura Tanganyika Aquiraz Brazil; ^3^ Laboratório de Ictioplâncton, Nupélia (Núcleo de Pesquisas em Limnologia, Ictiologia e Aquicultura), Programa de Pós‐graduação em Ecologia de Ambientes Aquáticos Continentais (PEA), Centro de Ciências Biológicas (CCB), Universidade Estadual de Maringá (UEM) Maringá Brazil; ^4^ Laboratório de Ictiologia Museu de Zoologia da Universidade Estadual de Londrina Londrina Brazil

**Keywords:** Amazon basin, early development, larvae, ontogeny, Ostariophysi

## Abstract

*Leporinus oliveirai* is a recently described species from Serra do Cachimbo, Pará State, Brazil, notable for its small size and vibrant coloration. This species has been successfully reproduced in captivity and is commercially traded internationally. Herein, we describe the early development of *Leporinus oliveirai*, documenting its external morphology with photographs and illustrations. The ontogeny of *L. oliveirai* is compared with that of other anostomids for which ontogenetic data are available, and developmental patterns are described for the family. In Anostomidae, the critical processes associated with the transition to exogenous feeding occur within the first 5 days post‐hatching. Fin formation occurred in two distinct phases: the anal, dorsal, caudal, and adipose fins developed first, followed by the pelvic and pectoral fins. The sequence of fin ossification in anostomids is generally consistent with patterns described for other Characiformes. Additionally, a marked increase in growth rate and indications of allometric growth were observed following notochord flexion.

## Graphical abstract

Herein, we describe the early development of *Leporinus oliveirai*, a recently described species, documenting its external morphology with photographs and illustrations. The ontogeny of *L. oliveirai* is compared with other anostomids and developmental patterns are described for the family. In Anostomidae, the critical processes associated with the transition to exogenous feeding occur within the first 5 days post‐hatching, the sequence of fin ossification is consistent with patterns described for other Characiformes and a marked increase in growth rate and allometric growth were observed following notochord flexion.

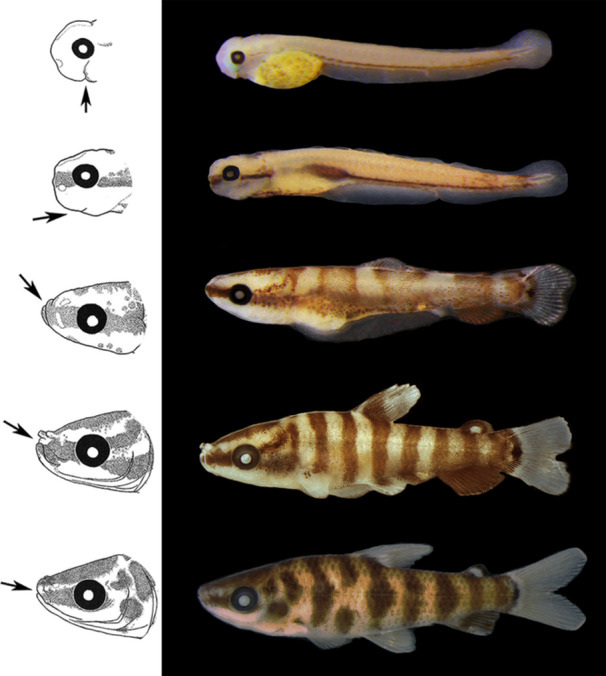

## 1 Introduction

The Neotropical fish fauna is among the most diverse in the world and our understanding of this biodiversity is continuously expanding. Over the past decade, an average of 100 new fish species have been described annually (Reis et al. 2016; Birindelli and Sidlauskas 2018; Albert et al. 2020). However, despite significant efforts to document and understand this diversity, freshwater fish populations have been experiencing a sharp decline. Over the past 50 years, 80% of freshwater vertebrate populations have decreased due to accelerated environmental changes, compounded by increasing pollution and unsustainable resource exploitation (Darwall et al. 2018).

Given this scenario of accelerated biodiversity loss, it is increasingly urgent to expand knowledge about the Neotropical ichthyofauna, including its taxonomic diversity, ecology and evolutionary relationships. A relevant example is the family Anostomidae, one of the most diverse groups within Characiformes, which comprises 148 valid species across 17 genera (Sidlauskas et al. 2025). Members of Anostomidae are distinguished by their unique dentition, characterized by three or four large incisiform teeth in both premaxilla and dentary bones (Garavello and Britski 2003). The genus *Leporinus* (Agassiz 1829), is the most species‐rich within the family, with approximately 80 valid species distributed across all major South American basins (Fricke et al. 2023; Toledo‐Piza et al. 2024). This genus is characterized by a terminal or subterminal mouth, four teeth on the dentary, and three or four unicuspid, incisiform teeth on the premaxilla (Sidlauskas and Birindelli 2017). Despite these shared traits, molecular phylogenies have revealed the genus to be polyphyletic, indicating that its current taxonomic composition does not accurately reflect evolutionary relationships (Ramirez et al. 2016; Birindelli et al. 2020a; Sidlauskas et al. 2025).

Knowledge of the early development of the Anostomidae is still limited, and according to Reynalte‐Tataje et al. (2020), fewer than 20% of species have been studied, with most data derived from species frequently reproduced in captivity (e.g., Sanches et al. 2001; Sampaio and Sato 2009; Sousa et al. 2014; Ferreira 2015) or occasionally encountered during reservoir monitoring (e.g., Nakatani et al. 2001; Orsi et al. 2016). Ontogenetic studies include species such as *Leporinus agassizii* (Steindachner 1876; Ferreira 2015), *L. amblyrhynchus* (Garavello and Britski 1987; Orsi et al. 2016), *L. piau* Fowler 1941 (Borçato et al. 2004; Sampaio and Sato 2009), *L. friderici* (Bloch 1794) (Sanches et al. 2001; Nakatani et al. 2001; Orsi et al. 2016), *Hypomasticus steindachneri* (Eigenmann and Ogle 1907) (Souza et al. 2017), *Megaleporinus elongatus* (Valenciennes and Cuvier 1850) (Sousa et al. 2014; Perrotti et al. 2019), *M. obtusidens* (Valenciennes 1837) (Santos and Godinho 1996; Nakatani et al. 2001; Sousa et al. 2014; Orsi et al. 2016), *M. piavussu* (Britski et al. 2012) (Nakatani et al. 2001), *Schizodon borellii* (Boulenger 1900) (Nakatani et al. 2001; Orsi et al. 2016) and *Schizodon fasciatus* (Spix and Agassiz 1829) (Nakatani et al. 2001). Studies using specimens collected entirely on natural habitats are even rarer, with four notable examples: Santos (1980, 1982) investigated *L. fasciatus* (Bloch 1794), *M. trifasciatus* (Steindachner 1876), and *Rhytiodus microlepis* (Kner 1858); Machado‐Evangelista et al. (2015) studied *L. reticulatus* (Britski and Garavello 1993), primarily examining changes in mouth morphology associated with dietary adaptations, and lastly, Santos et al. (2024) described in detail the early ontogeny of *R. microlepis*. This literature reveals a significant gap in the knowledge of the early development of anostomids, which hinders a complete understanding of the reproductive biology and survival strategies of the group. Furthermore, a detailed understanding of anostomid ontogeny is useful for the identification of larvae collected in the natural environment, enabling species surveys, the inventory of spawning and natural breeding grounds and for studies of ichthyoplankton ecology (Nakatani et al. 2001).

**FIGURE 1:**
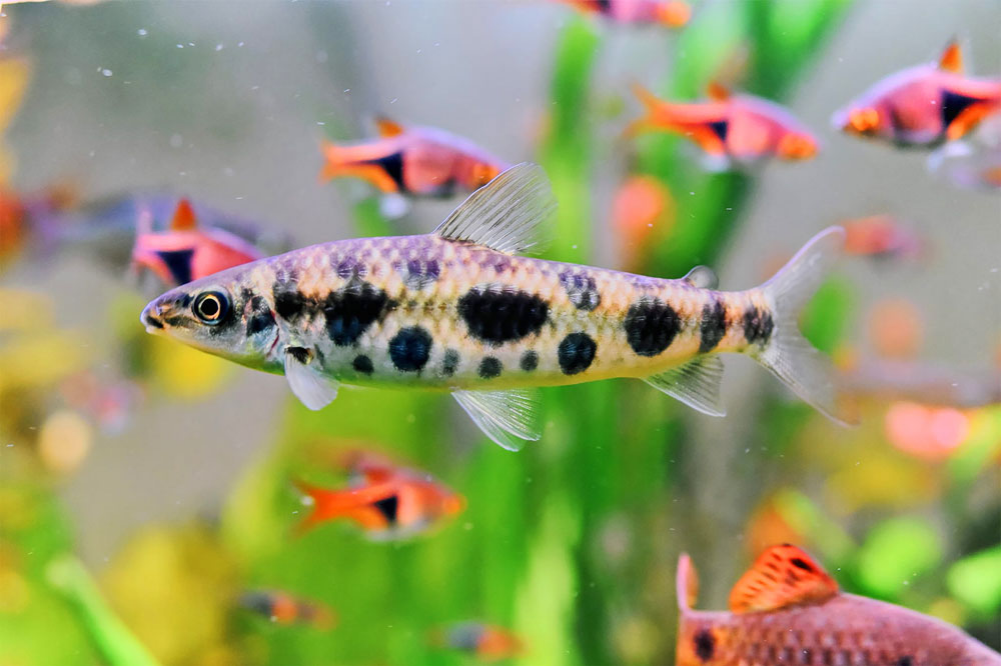
Live specimen of *Leporinus oliveirai* (Photo by Kan Paloo).

*Leporinus oliveirai* (Ito et al. 2023), is a small‐sized species recently described as endemic to Serra do Cachimbo, inhabiting the Rio Braço Norte, a tributary of the Teles Pires River within the Tapajós River basin, in Pará State, Brazil (Figure 1). In recent years, this species has been successfully reproduced in captivity and has since gained popularity in both national and international aquarium trade markets. The ontogeny of the skeleton of this species was recently described (Boaretto et al. 2025), representing the first description of the skeletal ontogeny in an anostomid fish. In this study, we provide a detailed description and illustrations of the external morphology during the early development of *L. oliveirai*. By analyzing its ontogenetic stages and comparing them with available data from other anostomid species, we aim to improve the identification of larval stages and contribute to the broader understanding of developmental patterns in Anostomidae.

## 2 Material and Methods

### 2.1 Source of Biological Material

The procedures for reproduction and preservation of the studied specimens were previously described in Boaretto et al. (2025). All vouchers are deposited at Museu de Zoologia da Universidade Estadual de Londrina (MZUEL 20844).

### 2.2 Analysis of Biological Material

**FIGURE 2:**
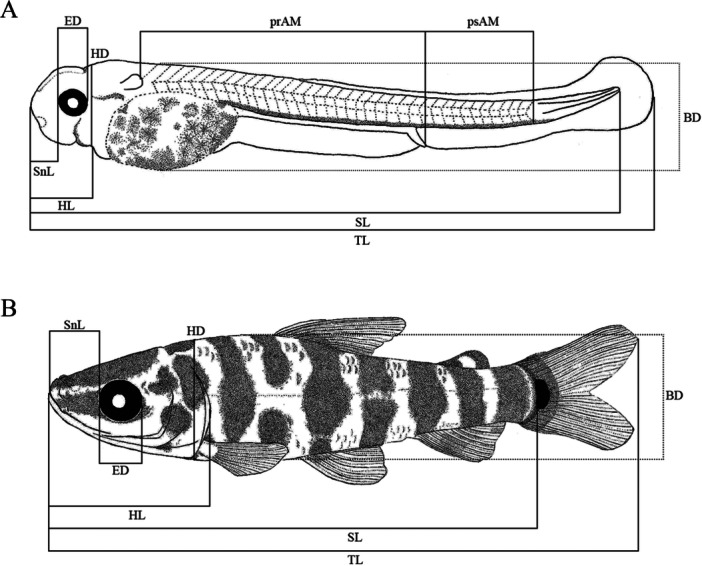
Morphometric measurements and counts from (A) larvae and (B) juveniles of *Leporinus oliveirai*. Abbreviations: total length (TL), standard length (SL), snout length (SnL), eye diameter (ED), head length (HL), head depth (HD) and body depth (BD), pre‐anal myomeres (prAM), post‐anal myomeres (psAM).

Our classification followed the stablished larval (yolk‐sac, preflexion, flexion, postflexion) and juvenile stages, according to Nakatani et al. (2001) modified from Ahlstrom et al. (1976). Meristic and morphometric data followed Nakatani et al. (2001) and Birindelli and Britski (2013). A total of 60 specimens were photographed and measured with a digital camera DFC295 attached to a stereomicroscope Leica M205A. The following measures were taken (Figure 2): total length (TL), standard length (SL), snout length (SnL), eye diameter (ED), head length (HL), head depth (HD) and body depth (BD). Analyzed meristics were pre‐anal myomeres (prAM), post‐anal myomeres (psAM) and, when present, scales on the lateral line (ScLL). Body relationships (Biometric characteristics based on proportions relative to standard length and head length) were established using the criteria described by Leis and Trnski (1989) modified by Nakatani et al. (2001). Patterns of body growth were evaluated through regression analysis, where the morphometric variables (dependent variables) were plotted against SL and HL (independent variables) using a simple linear regression model performed in the PAST ver.4.10 software (Hammer et al. 2001). For each stage of development, specimen illustrations were made with technical black pens, line size 0.01, 0.03 and 0.05 following the technique described by Shibatta (2017).

## 3 Results

### 3.1 Larval Development

**FIGURE 3:**
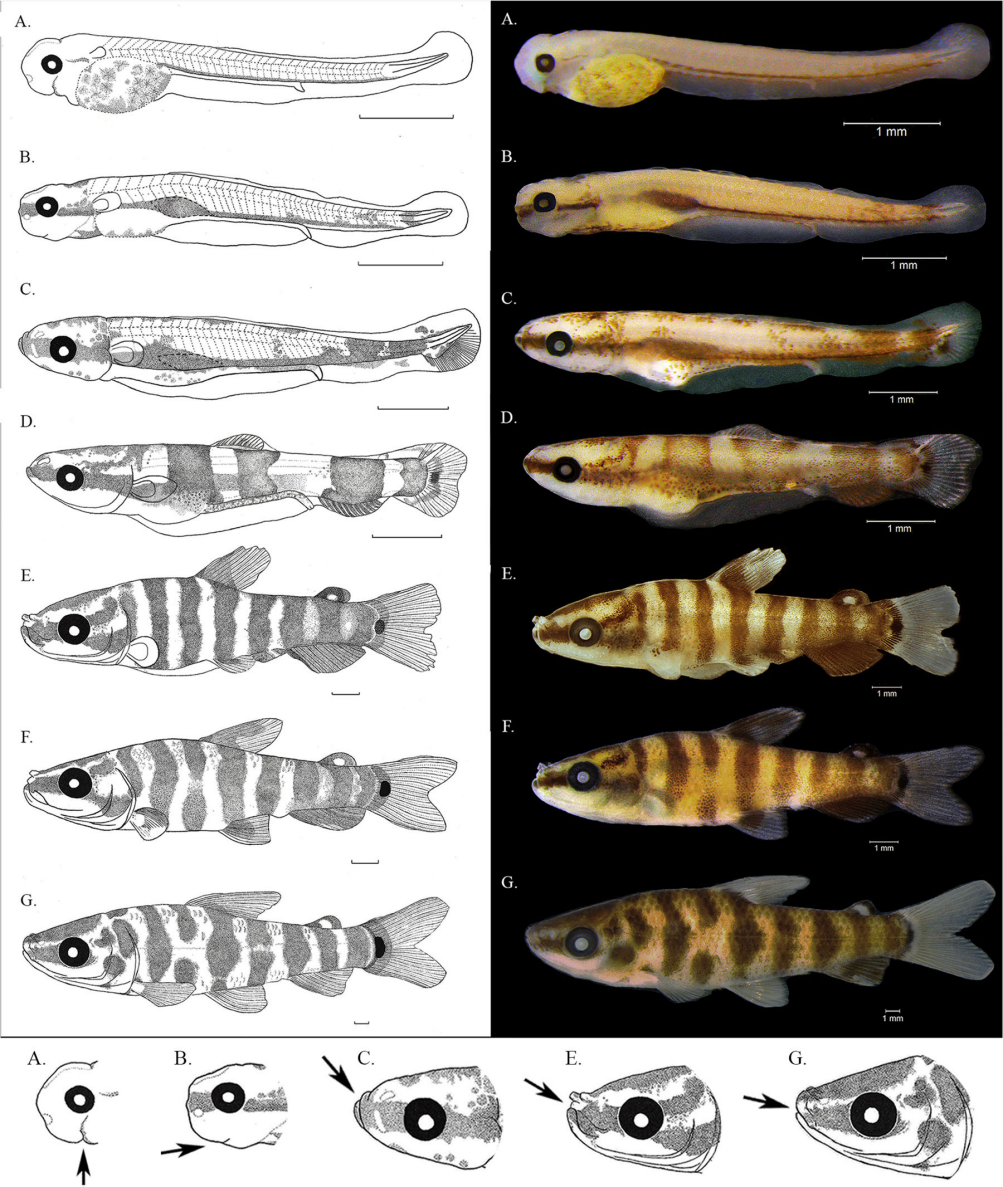
Larval and juvenile development of *Leporinus oliveirai*. Lower panel highlights the ontogenetic shift in mouth cleft position (indicated by black arrow). (A) yolk‐sac larvae (5.02 mm), (B) preflexion (5.88 mm), (C) flexion (6.63 mm), (D) flexion (7.52 mm), (E) post‐flexion (14.42 mm), (F) post‐flexion (16.01 mm) and (G) juvenile (26.59 mm). Scale bar = 1 mm.

**FIGURE 4:**
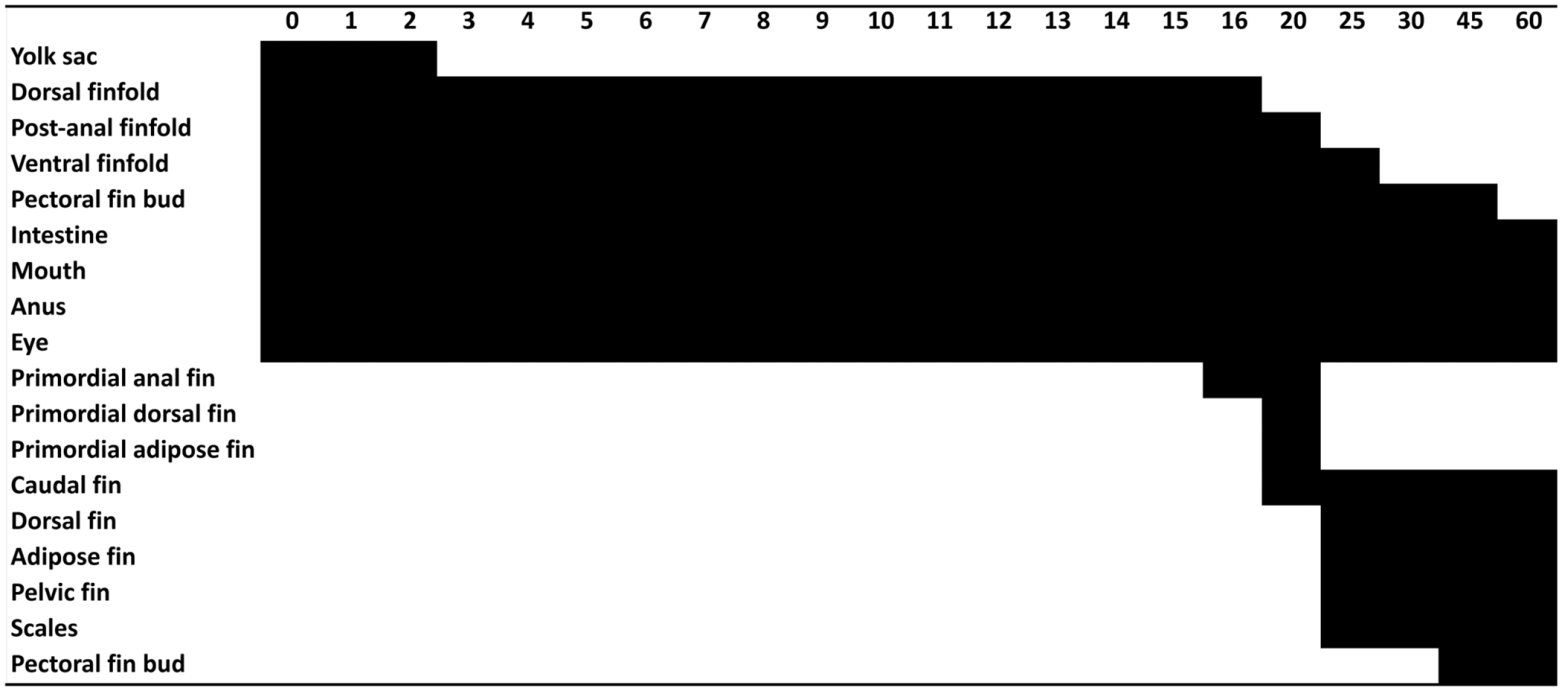
Sequence of ontogenetic events of *Leporinus oliveirai*. Black bars along horizontal axis represents the day's post hatching at which a particular structure was observed in all individuals.

**Yolk‐sac stage (days 0–1,** Figure 3a **).** This stage spans from hatching to the onset of exogenous feeding, characterized by partial or complete eye pigmentation and the opening of the mouth and anus. The standard length (SL) of the analyzed specimens ranged from 4.82 to 5.28 mm. The body is elongated, with the notochord clearly visible along its entire length (Figure 3a). The pectoral fin bud is present. A finfold is present, being more developed ventrally than dorsally. The intestine is long and discernible, extending to the posterior portion of the body. The branchial opening is apparent but not pronounced. Although the mouth and anus are visible, they are not open at this stage. The mouth is positioned ventrally, beneath large, pigmented eyes located directly above it. The yolk‐sac is prominent throughout this stage, gradually decreasing in size (Figure 4). Dendritic chromatophores are observed across the surface of the yolk sac, being predominantly concentrated in the ventral region. A distinct dark stripe extends from the tip of the snout to the end of the notochord, interrupted briefly by the yolk‐sac, and forms a vertically compressed dark blotch immediately posterior to the sac. Pigmentation begins in the posterodorsal region of the head and becomes larger in the anteroventral region of the body as the yolk‐sac diminishes. Additionally, the posterior region of the body, near the tip of the notochord, starts to show pigmentation during this stage. While myomeres are present, they are only faintly visible, making their precise count unfeasible at this stage.

**Preflexion stage (days 2–9,** Figure 3b **).** This stage extends from the onset of mouth and anus functionality and eye pigmentation to the initiation of notochord flexion. The standard length of specimens analyzed ranged from 5.09 to 6.08 mm. The yolk‐sac remains visible but progressively reduces in size until it disappears at 5.33 mm (Figure 3b). Early in the stage, the mouth is open and ventrally positioned, but it gradually shifts towards a terminal position and later becomes slightly upturned. Nostrils simple and snout rounded. Operculum present. The swim bladder is inflated and visible. Anus opened. The pectoral fin bud starts to increase at size, without the presence of rays in this stage. The notochord displays no signs of flexion and retains a horizontally positioned posterior tip. The longitudinal stripe darkens and widens, becoming more pronounced, particularly in the head region. The previously compressed dark blotch enlarges vertically, particularly around the swim bladder. The number of chromatophores increases across the body, especially in the post‐anal region, where two distinct areas of concentrated pigmentation begin to form, which will later develop into the two vertical bars of the caudal region. Additionally, the number of chromatophores increases in the posterodorsal region of the head. Pigmentation also appears over the intestine during this stage. In the later days of the stage (Day 9, around 6 mm SL), a few chromatophores become noticeable on the dorsal part of the body and in the finfold around the posterior tip of the notochord. A total of 35–37 myomeres were observed, with 22–24 pre and 13–14 post‐anal.

**Flexion stage (Days 10–20,** Figure 3c,d **).** This stage spans from the onset of notochord flexion to its complete flexion; it also generally involves at least partial formation of the dorsal and anal fin rays, as well as the emergence of the pelvic fin bud. The standard length of specimens analyzed ranged from 6.52 to 8.69 mm. The mouth cleft adopts a gently upward orientation. The tip of the notochord begins to shift dorsally, and the supporting elements of the caudal fin begin to develop (Figure 3c,d), with the first rays becoming visible. Complete flexion was observed in specimens at 20 days of age (Between 8.38 and 8.69 mm SL) when also the rays of the dorsal and anal fins begin to develop, and the adipose fin also appears. The pectoral fin bud increases in size. The finfold remains visible and supports the formation of the median fins. The swim bladder becomes gradually less visible due to the decrease of body transparency. After flexion, the notochord progressively becomes less distinguishable, but it remains visible on the upper portion of the caudal fin. Pigmentation increases across the entire body. The anterior portion of the longitudinal stripe, extending from the snout tip to the anterior margin of the eye, becomes more defined and darker. The posterior portion of the stripe, from the posterior eye margin to the caudal fin, gradually fades, along with the compressed blotch, which nearly disappears from the body, leaving only traces on the head in the later days of the stage (Between 8.38 and 8.69 mm SL). Three vertical dark bars begin to form at this stage: the first immediately posterior to the head, the second before the origin of the developing dorsal fin, and the third below the posterior portion of the developing dorsal fin. The five vertical bars, including the two in the post‐anal region, become well‐defined in the later days of the stage. A pigmented semicircular stripe forms at the base of the caudal fin, and a distinct, conspicuous dark spot appears at the midlateral point of the body. The developing dorsal, anal, and adipose fins also acquire pigmentation during the later days of the stage. A total of 36 or 37 myomeres were counted, with 23 or 24 myomeres located anteriorly to the anal opening and 13 posteriorly.

**Postflexion stage (days 25–45,** Figure 3e,f **).** This stage spans from the complete flexion of the notochord to the complete formation of the pectoral fin rays and the complete absorption of the finfold. The standard length of specimens analyzed ranged from 12.36 to 16.49 mm. The body becomes taller, and its overall shape starts to resemble that of a juvenile, making the larvae distinctly different from those in earlier stages (Figure 3e,f). The head increases significantly in size, occupying approximately one‐third of the standard length, becoming more distinct from the rest of the body, with a clear opercular boundary. Some late‐stage specimens exhibited a lateral line with 36 scales. Both anterior and posterior nostrils are visible, with the anterior pair becoming tubular early in this stage (12.36 mm SL). The tip of the notochord is almost indistinguishable at this stage, restricted to a single narrow line along the upper lobe of the caudal fin. The pelvic fin bud appears (12.36 mm SL), along with the anal opening. During the later days of the stage (14.29 mm SL), the finfold is completely absorbed, rays begin to appear at the pectoral fin bud (14.12 mm SL) and scales emerge on the dorsal portion of the body (14.29 mm SL), (Figure 4). The dorsal, pelvic, anal, and adipose fins develop increased pigmentation during this stage. The horizontal stripe is now only present on the head. The second, third, and fourth vertical bars become divided, each originating two new bars. A total of eight dark vertical bars are now visible, extending from the posterior margin of the head to the caudal fin peduncle. By the end of the stage, the second, fourth, fifth, and sixth bars become slightly enlarged, while the third bar is interrupted at midlateral points. The midlateral stripe persists, extending from the snout tip to the posterior margin of the opercle, joining the first vertical bar and forming an inverted L‐shaped blotch. An additional stripe becomes visible, ascending from the posterior margin of the orbit to the dorsal portion of the first vertical bar. Due to the intense pigmentation and increased skin thickness, the myomeres could no longer be counted. At the end of the stage, all fins are completely formed, and the ossification sequence is as follows: caudal (i,8,9,i), anal (iii,8), dorsal (ii,10), pelvic (i,8), and pectoral (i,13–16).

**Juvenile development (day 60,** Figure 3g **).** At this stage, larval development is complete, but sexual maturity has not yet been reached. This stage is marked by the presence of all the fin rays and scales covering the entire body (Figure 4). The standard length of specimens analyzed ranged from 26.59 to 26.98 mm. The body proportions are similar to those of an adult individual, with an elongated, fusiform body and a relatively smaller head (Figure 3g). The mouth has migrated from an upturned to a more terminal position. The first pair of tubular nostrils become proportionally smaller and less visible. The specimens exhibit 36–37 scales along the lateral line and 12 scales around the caudal peduncle. The overall pigmentation differs from that of the earlier larval stages, although it is not yet identical to that of adult specimens. The second, fourth, and sixth enlarged vertical bars become distinctly round and enlarged at midlateral points, forming the midlateral blotches characteristic of adults, though they are not completely separated from the bars. The first and third vertical bars become fully separated, while the sixth bar only begins the process of separation. Pigmentation in the dorsal, anal, and pelvic fins diminishes, and the adipose fin becomes pigmented at the distal and proximal margins, as observed in adults. The pigmented semicircular stripe at the base of the caudal fin and the conspicuous midlateral dark spot fade significantly. The dorsal portion of the head darkens, and the midlateral stripe is interrupted near the posterior margin of the orbit, resulting in a separate dark blotch on the opercle that persists in adults. The upper additional stripe becomes a conspicuous dark spot located at the posterodorsal margin of the infraorbital series, which remains visible in adults.

### 3.2 Morphometric Relationships and Growth

From yolk‐sac larvae to the juvenile period, the specimens exhibited morphometric variation, ranging from elongated to moderately tall body, following the classification proposed by Leis and Trnski (1989) (BD 14.91%–27.13% SL) (Table 1). Relative head size varied from small to large (HL 11.04%–33.11% SL), initially small during the yolk‐sac stage (11.04%–16.57% SL) and progressively increasing through the preflexion (18.6%–24.19% SL), flexion (22.23%–25.02% SL), and postflexion stages (30.44%–33.11% SL), before slightly decreasing in juveniles (23.30%–23.77% SL). Relative eye size also varied from small to large (ED 22.85%–39.58% HL), initially large during the yolk‐sac stage (27.25%–39.58% HL), decreasing in the subsequent stages (preflexion: 22.85%–30.95% HL, flexion: 23.02%–32.93% HL, postflexion: 27.66%–29.67% HL), before increasing again in juveniles (35.42%–38.70% HL). Other proportions, such as relative snout length and head depth, also exhibited significant variation throughout development (SnL 20.86%–44.88% HL, HD 57.87%–118.94% HL). Relative head length, body depth, and snout length increased in proportion during development, while relative head depth slightly decreased in proportion.

**TABLE 1:** Values of minimum (Min), maximum (Max), mean length and standard deviation (sd) of morphometric and meristic variables from larvae and juveniles of *Leporinus oliveirai*.

	Larval period									
	Yolk‐sac larva (*n* = 6)		Pre‐flexion (*n* = 24)		Flexion (*n* = 20)		Post‐flexion (*n* = 6)		Juvenile (*n* = 4)	
Measurements (mm)	Mean ± sd	Min‐Max	Mean ± sd	Min‐Max	Mean ± sd	Min‐Max	Mean ± sd	Min‐Max	Mean ± sd	Min‐Max
Total length	5.27 ± 0.17	5.14–5.52	6.14 ± 0.3	5.69–6.902	7.75 ± 0.96	6.84–9.57	17.25 ± 2.53	14.32–20.23	31.89 ± 0.59	31.31–32.72
Standard length	5.07 ± 0.20	4.82–5.28	5.79 ± 0.39	5.09–6.58	7.37 ± 0.77	6.52–8.69	14.36 ± 1.82	12.36–16.49	26.82 ± 0.16	26.59–26.98
Snout length	0.22 ± 0.01	0.20–0.23	0.31 ± 0.05	0.23–0.41	0.51 ± 0.08	0.41–0.67	1.51 ± 0.24	1.23–1.82	2.76 ± 0.05	2.72–2.84
Eye diameter	0.22 ± 0.01	0.21–0.25	0.33 ± 0.03	0.23–0.39	0.47 ± 0.08	0.33–0.61	1.29 ± 0.18	1.08–1.51	2.36 ± 0.10	2.22–2.44
Head length	0.70 ± 0.14	0.53–0.87	1.27 ± 0.16	1.01–1.48	1.73 ± 0.23	1.47–2.17	4.56 ± 0.74	3.77–5.46	6.31 ± 0.01	6.28–6.33
Head depth	0.66 ± 0.02	0.63–0.69	0.85 ± 0.05	0.75–0.97	1.13 ± 0.18	0.91–1.44	3.13 ± 0.54	2.64–3.86	6.11 ± 0.09	6.0–6.2
Body depth	0.98 ± 0.01	0.95–0.99	0.96 ± 0.05	0.85–1.05	1.30 ± 0.27	0.99–1.72	3.59 ± 0.66	2.86–4.40	7.18 ± 0.09	7.13–7.32
**Proportions (%)**										
HL/SL	13.8 ± 2.38	11.04–16.57	21.95 ± 1.83	18.6–24.19	23.46 ± 0.82	22.23–25.02	31.67 ± 1.17	30.44–33.11	23.53 ± 0.19	23.30–23.77
BD/SL	19.36 ± 1.01	18.15–20.37	16.62 ± 0.87	15.01–18.55	17.51 ± 1.98	14.91–20.66	24.90 ± 1.45	23.16–26.72	26.77 ± 0.26	26.52–27.13
SnL/HL	33.13 ± 7.52	24.10–41.65	24.34 ± 2.12	20.86–28.65	29.74 ± 1.68	27.02–32.66	33.25 ± 1.41	31.12–35.31	43.75 ± 0.81	43.10–44.88
ED/HL	33.20 ± 5.0	27.25–39.58	26.62 ± 2.11	22.85–30.95	27.17 ± 2.31	23.02–32.93	28.52 ± 0.79	27.66–29.67	37.38 ± 1.47	35.42–38.70
HD/HL	97.69 ± 16.7	79.58–118.94	67.82 ± 7.6	57.87–84.47	65.48 ± 4.0	60.40–77.76	68.65 ± 3.21	63.46–71.51	96.87 ± 1.79	94.96–98.80
**Number of myomeres**										
	**Mode**	**Min‐Max**	**Mode**	**Min‐Max**	**Mode**	**Min‐Max**	**Mode**	**Min‐Max**	**Mode**	**Min‐Max**
prAM	DV	DV	23	22–24	24	23–24	NV	NV	NV	NV
psAM	DV	DV	13	13–14	13	13	NV	NV	NV	NV
total	DV	DV	36	35–37	37	36–37	NV	NV	NV	NV
**Number of scales**										
ScLL	NV	NV	NV	NV	NV	NV	36	36	36	36–37

Abbreviations: BD, body depth; DV, difficult visualization; ED, eye diameter; HD, head depth; HL, head length; n, number of individuals;NV, not visible; prAM, pre‐anal myomeres; psAM, post‐anal myomeres; ScLL, scales on the lateral line; SL, standard length; SnL, snout length.

**FIGURE 5:**
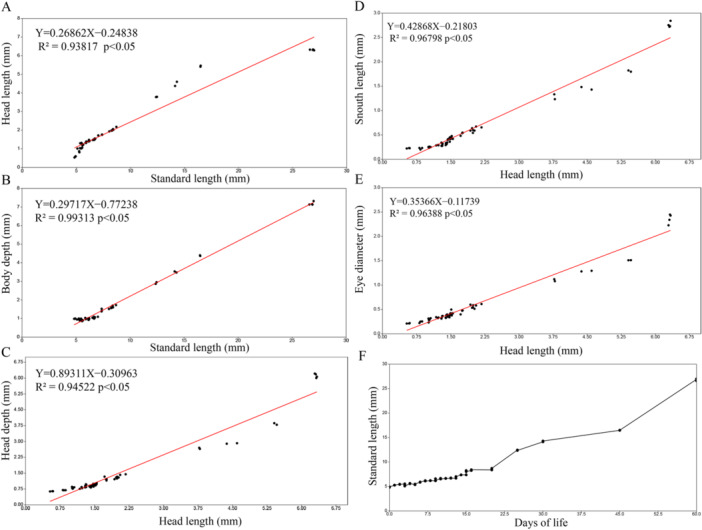
Morphometric correlation (mm) between the standard length and (A) head length, (B) body depth, and between head length and (C) head depth, (D) snout length and (E) eye diameter of *Leporinus oliveirai*. Scatter plot of the correlation between the day's post hatching and (F) length of *Leporinus oliveirai*.

The morphometric variables presented an overall high value on the coefficient of determination (R²), which indicates how much the independent variable explains the dependent variable, in this case, how strong was the linear relationship between the variables. Relations between head length with standard length and between head depth, snout length and eye diameter with head length presented a general strong linear relationship, with R² ranging from 0.93 to 0.96 (Figure 5a–e). The analysis pointed out that the variables tend to distance themselves from the linear growth tendency after flexion period, between 20 and 25 days of life, usually after reaching about 10 mm SL and 3.75 mm HL, these results indicate that from this period onwards, these variables show strong signs of allometric growth. However, the relation between body depth and standard length presented an strong linear relationship, with R² value of 0.99, indicating that during the entire developmental period, body depth presented a continuous linear growth along with body length. All linear regressions presented an *p*‐value < 0.05, indicating statistical significance for these relations. The scatter plot (Figure 5f) depicts the relationship between the days of life and length, showing a constant growth rate until around 20–22 days of life, when an exponential growth begins and continues until the juvenile period, as the beginning of allometric growth, the beginning of exponential growth also occurs after flexion of the notochord.

## 4 Discussion

We provided one of the first detailed descriptions of the ontogeny of the external morphology of an anostomid species, contributing to the still limited knowledge of the development patterns within the family. *Leporinus oliveirai* differ from other anostomids primarily in the latter stages of development, and our findings can assist larval identification at the family level. From the postflexion stage to the juveniles, the characteristic vertical bars common to all anostomids gradually give way to a series of midlateral blotches, similar to the pigmentation pattern seen in adults. Nevertheless, *L. oliveirai* larvae exhibit typical anostomid traits, such as a fusiform body shape, a long intestine and initially simple nostrils that become double and tubular during ontogeny (Sanches et al. 2001; Santos et al. 2024).

The number of myomeres observed in *L. oliveirai* (35–37) is consistent with previously reported in the genus (35–37 in *L. friderici* and 38 in *L. amblyrhynchus*), slightly lower than those in the genus *Megaleporinus* (36–38 in *M. piavussu* and 35–40 in *M. obtusidens*) and markedly lower than in *Rhytiodus microlepis* (49–50). In *Schizodon*, myomere count varies notably among species, 35–37 in *S. borelli*, 35–39 in *S. fasciatus*, and 38–43 in *S. nasutus*. These differences may be associated with body shape, as more elongated species, such as *R. microlepis*, show a greater number of myomeres. Within *Leporinus*, *L. oliveirai* is distinguished by a lower number of pre‐anal myomeres (22–24 vs. 25–27 in the other species).

Comparing species of anostomids which have detailed descriptions of the larval development (Table 2), it is noticeable that major processes of the early stages (beginning of exogenous feeding, eye pigmentation, formation of pectoral fin bud, mouth opening and absorption of the yolk sac) occur between 1 and 5 days post‐hatching. The only exception is *M. elongatus*, which exhibits a relatively delayed yolk sac absorption at 7 days post‐hatching (Perrotti et al. 2019). These processes occurred in specimens measuring between 3.76 and 5.45 mm SL.

**TABLE 2:** Sequence of onset of ontogenetic events of *Leporinus oliveirai* and other members of Anostomidae, with time of earliest appearance in day's post hatch (dph) and smallest specimen in which the developmental event was observed.

Stage	Onset of ontogenetic events	*Leporinus oliveirai*	*L. friderici*	*L. piau*	*Megaleporinus elongatus*	*M. obtusidens*	*M. piavussu*	*Rhytiodus microlepis*	*Schizodon borelli*	*S. fasciatus*	*S. nasutus*
**Yolk‐sac**	Eye black pigmented	1 dph (4.82 mm SL)	4.15 mm SL	2 dph (4.70 mm SL)	3 dph (4.51 mm SL)	4.60 mm SL	3.95 mm SL	ad	4.20 mm SL	4.20 mm SL	4.45 mm SL
**Pre‐flexion**	Formation of pectoral bud	2 dph (5.45 mm SL)	4.10 mm SL	5 dph (5.30 mm SL)	5 dph (4.50 mm SL)	4.45 mm SL	3.95 mm SL	ad	4.15 mm SL	3.76 mm SL	4.15 mm SL
	Mouth opening	ad	ad	4 dph (5.34 mm SL)	5 dph (5.09 mm SL)	ad	ad	ad	ad	ad	
	Yolk sac fully absorbed	2 dph (5.42 mm SL)	4.73 mm SL	4 dph (5.34 mm SL)	7 dph (5.17 mm SL)	4.87 mm SL	5.00 mm SL	ad	ad	4.20 mm SL	5.20 mm SL
**Flexion**	Formation of caudal‐fin rays	16 dph (8.42 mm SL)	7.09 mm SL	ad	ad	8.72 mm SL	ad	7.20 mm SL	ad	7.00 mm SL	7.36 mm SL
	Formation of anal‐fin rays	16 dph (8.42 mm SL)	7.09 mm SL	ad	ad	8.72 mm SL	ad	11.31 mm SL	ad	9.24 mm SL	10.00 mm SL
	Formation of dorsal‐fin rays	20 dph (8.32 mm SL)	7.09 mm SL	ad	ad	8.72 mm SL	ad	11.31 mm SL	ad	9.24 mm SL	7.91 mm SL
	Formation of adipose fin	20 dph (8.69 mm SL)	8 mm SL	ad	ad	8.72 mm SL	ad	11.31 mm SL	ad	9.24 mm SL	10.00 mm SL
**Post‐flexion**	Formation of pelvic‐fin rays	25 dph (12.36 mm SL)	16.57 mm SL	ad	ad	10.38 mm SL	12.71 mm SL	13.08 mm SL	ad	10.86 mm SL	13.00 mm SL
	Uppermost pectoral‐fin rays	45 dph (16.47 mm SL)	16.57 mm SL	ad	ad	ad	ad	ad	ad	16.00 mm SL	13.75 mm SL
**Reference**		Present study	Sanches et al. (2001); Nakatani et al. (2001)	Borçato et al. (2004)	Sousa et al. (2014); Perrotti et al. (2019)	Nakatani et al. (2001); Sousa et al. (2014)	Nakatani et al. (2001)	Santos et al. (2024)	Nakatani et al. (2001)	Nakatani et al. (2001)	Nakatani et al. (2001)

Abbreviation: ad, absent data.

Fin development in *L. oliveirai* occurred in two periods: the anal, dorsal, caudal and adipose fins formed between 7 and 11.31 mm SL, while the pelvic and pectoral fins appeared slightly later, between 10.38 and 16.57 mm SL. The fin sets have different origins, with the finfold giving rise to the median fins, while the paired fins develop from distinct mesodermal buds. The sequence of ossification of the fins in anostomids is overall similar to that described for other Characiformes (Nakatani et al. 2001). Additionally, our findings corroborate a pattern previously reported for *R. microlepis* (Santos et al. 2024), an abrupt increase in growth rate and the presence of signs of allometric growth after notochord flexion.

Mouth position is a trait of recurrent discussion within Anostomidae, as many species and clades are recognized based on this characteristic, which is related to the ecological niche and feeding habits of the species. In a study on the diet and ecomorphology of *Leporinus reticulatus* (Machado‐Evangelista et al. 2015), smaller individuals (16.9 and 34.6 mm SL) exhibited a terminal mouth position, which gradually shifted to a subterminal position as development progressed. This contrasts with the pattern observed in *R. microlepis*, which maintained a terminal or slightly upturned mouth through most of its documented development (4.31–44.09 mm SL). A similar pattern was also reported for *S. fasciatus* (Nakatani et al. 2001).

In *L. oliveirai*, a transition from slightly upturned to terminal mouth position was observed from flexion stage to the juvenile period (6.52–32.72 mm SL, Figure 3). This developmental pattern has also been reported in other species of *Leporinus* and *Megaleporinus* species. Given the subterminal mouth observed in adults and the phylogenetic proximity among these species (Ito et al.^2023^), it is expected that *L. oliveirai* undergoes the same ontogenetic shift described for *L. reticulatus*. This suggests that such a development pattern may be a common feature among *Leporinus* species that exhibit a subterminal mouth.

Although mouth position remains an important trait for the identification and phylogenetic relationships of anostomids, a recent study investigating phenotypic plasticity (Bonini‐Campos et al. 2019) demonstrated that *Megaleporinus macrocephalus* specimens raised under different environmental and feeding conditions developed different morphotypes, with significant variation in mouth position. These findings suggest that environmental conditions play a considerable role in mouth development, specifically during the early stages. This phenotypic plasticity may be key to understanding the high morphological diversity observed within Anostomidae.

In summary, our study contributes to the understanding of early development in Anostomidae and Characiformes, providing insights into their ontogeny. The data obtained not only corroborate development patterns previously reported for the family but also provide detailed descriptions of morphological and pigmentation traits that can help the identification of specimens at both family and genus levels. Furthermore, expanding ontogenetic research contributes to understand the developmental and evolutionary trajectories of key traits in anostomids.

## Author Contributions

**Ian Solon Bortoloci Ito:** conceptualization, data curation, formal analysis, writing – original draft, writing – review and editing, investigation, validation, visualization. **Mariana Pascoal Boaretto:** formal analysis, validation, writing – original draft, writing – review and editing, visualization. **Marcos Venturieri:** methodology, data curation, investigation, writing – original draft. **Andréa Bialetzki:** validation, visualization, writing – original draft, writing – review and editing, methodology. **José Luís Olivan Birindelli:** conceptualization, funding acquisition, project administration, resources, validation, writing – review and editing, supervision.

## 1 Peer Review

The peer review history for this article is available at https://www.webofscience.com/api/gateway/wos/peer-review/10.1002/jmor.70065.

## Data Availability

The data that support the findings of this study are available in the supporting material of this article.
